# Programmable DNA Nanosystem for Molecular Interrogation

**DOI:** 10.1038/srep27413

**Published:** 2016-06-07

**Authors:** Divita Mathur, Eric R. Henderson

**Affiliations:** 1 Department of Genetics, Development and Cell Biology, Iowa State University, Ames, IA 50011, USA; 2 Bioinformatics and Computational Biology Program, Iowa State University, Ames, IA 50011, USA; 3 College of Science, George Mason University, Fairfax, USA; 4 Center for Bio/Molecular Science and Engineering, Code 6900, U.S. Naval Research Laboratory, Washington, USA

## Abstract

We describe a self-assembling DNA-based nanosystem for interrogating molecular interactions. The nanosystem contains a rigid supporting dumbbell-shaped frame, a cylindrical central core, and a mobile ring that is coaxial with the core. Motion of the ring is influenced by several control elements whose force-generating capability is based on the transition of single-stranded DNA to double-stranded DNA. These forces can be directed to act in opposition to adhesive forces between the ring and the frame thereby providing a mechanism for molecular detection and interrogation at the ring-frame interface. As proof of principle we use this system to evaluate base stacking adhesion and demonstrate detection of a soluble nucleic acid viral genome mimic.

In addition to the iconic genetic code, nucleic acid contains an “engineering” code. Recent conceptual and methodological advances have culminated in the availability of tools and strategies for leveraging this engineering code to program DNA to spontaneously create a diverse array of two- and three-dimensional shapes^1^,^2^,^3^,^4^,^5^. These shapes can be imbued with information and function including algorithmic calculations^6^,^7^,^8^, single-molecule analyses^9^,^10^, therapeutics^11^,^12^,^13^, mechanical actuation^14^,^15^, and a host of other capabilities^16^,^17^,^18^,^19^. Programmable, responsive actuation of dynamic self-assembling nanodevices is a highly desirable attribute and several studies have demonstrated mechanical reconfiguration of DNA nanodevices by thermal motion^14^ and upon triggering by a target molecule^11^,^20^,^21^,^22^,^23^,^24^,^25^,^26^. The study described here focuses on improvements in these responsive nanosystems in terms of modularity and robustness while minimizing undesirable conformational changes upon actuation. The DNA nanosystem described here enhances the repertoire of molecular reporting systems^25^,^27^ and serves as a base platform for molecular interrogation with an embedded reporter system module that is compatible with a variety of molecular species.

In this study we harness the difference in persistence length (i.e., rigidity) of single-stranded (ssDNA) and double-stranded (dsDNA) DNA to elicit a defined physical state change in a self-assembling DNA nanosystem we have termed OPTIMuS (Oligo-Propelled Technology for Interrogating Molecular Systems; Fig. 1). This inducible state change can be used to interrogate user-programmed molecular interactions within the OPTIMuS platform. In this report, we demonstrate how OPTIMuS can be used to detect a soluble target molecule and assess the relative strength of a non-covalent (base stacking) molecular interaction.

## Results

### Construction and Principle of Operation

**Figure 1:**
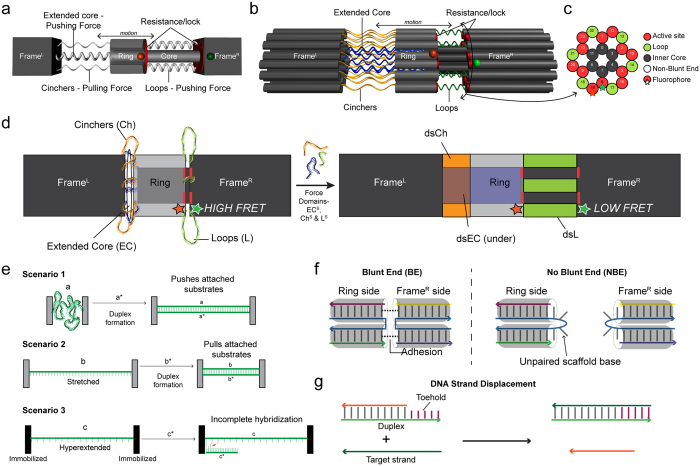
OPTIMuS operational principles. (**a**) A schematic illustrating the key mechanical components of OPTIMuS. A movable ring surrounds a cylindrical core that is anchored at both ends by “dumbbell” shaped frame elements. The ring is pushed and/or pulled away from frame^R^ by user-controlled ssDNA to dsDNA transitions, whereas “resistance” at the interface can obstruct ring movement. (**b**) A three-dimensional rendering of OPTIMuS showing the 24 helix bundle in the honeycomb lattice arrangement (Supplementary Figs 2 and 4). (**c**) A cross-sectional view of the ring/frame^R^ interface shows active sites, loops and the FRET reporter pair. (**d**) The idealized overall reconfiguration that can be elicited in OPTIMuS. On the left is the ground state (G) that has all force domains in single-stranded form (EC, Ch, L). Upon adding staples corresponding to them (EC^S^, Ch^S^, L^S^) the nanosystem reconfigures with a displaced ring position. Hybridization on the left-side (at EC and Ch) and the right-side (L) of the ring is reported by via FRET. (**e**) Depiction of the mechanism of force-induced motion by an ssDNA to dsDNA transition. The three force domains, EC, Ch and L are based upon the following scenarios. In scenario 1, hybridization of randomly coiled ssDNA creates a pushing force that increases the separation between attached substrates. In Scenario 2, pulling forces are created when a stretched ssDNA collapses into a short double helix upon hybridization, thereby bringing the substrates closer together. In scenario 3, which occurs when EC is formed before or simultaneously with Ch duplexes, the mechanically stretched ssDNA cannot form a duplex with its complement despite the favorable ΔG for the same molecules when stereochemically unconstrained. (**f**) A schematic of the blunt end and non-blunt end interactions between the coaxial helices of ring and frame^R^. Non-blunt ends are created by leaving eight scaffold bases at the crossover unhybridized. The resultant single-stranded region prevents base stacking and minimizes adhesive interaction between the duplexes. (**g**) Illustration of a toehold-mediated DNA strand displacement reaction.

The OPTIMuS platform is constructed following the principles of DNA origami, in which, specific ensembles of short oligonucleotides called “staples” are used to fold a large single-stranded “scaffold” into desired shapes (for details see Supplementary Information & Supplementary Fig. 2). The mechanical design of OPTIMuS is inspired by a system in which tunable “springs” exert pushing and pulling forces on a movable ring within a coaxial dumbell-shaped framework (Fig. 1a). These forces are opposed by introducing resistance at the interface between the mobile ring and the dumbbell frame. Finally, an embedded Förster Resonance Energy Transfer (FRET) system, in which one cyanine 3 (cy3) molecule is positioned on the frame and one cyanine 5 (cy5) molecule is on the ring, reports the relative position of the ring under various conditions (Supplementary Fig. 9). The main components of OPTIMuS are described in Fig. 1b.

The force elements are single-stranded scaffold domains that undergo structural change upon hybridizing to their complementary staple strands. Single-stranded DNA, an entropically elastic polymer with a formal contour length of 0.7 nm/base and persistence length of about 5 nm^28^, transitions into a rigid double-helix of 0.34 nm/bp contour length and 50 nm persistence length upon hybridizing with its complimentary strand^29^ (Fig. 1e). If the ends of an ssDNA molecule are tethered to two substrates, the relative distance between the substrates can be altered due to the internal reconfiguration concomitant with duplex formation (Fig. 1e Scenario 1 and 2). This spatial change can be exploited for applications in sensing^30^,^31^ and, potentially, molecular force/energy measurements. Importantly, although somewhat counterintuitive, if these substrates are immobilized, causing the ssDNA to be fixed at its full extenstion limit (≥0.7 nm/base), duplex formation becomes stereochmically inhibited despite the favorable ΔG of the reaction (Fig. 1e Scenario 3).

Three kinds of scaffold domains in OPTIMuS use the aforementioned phenomena to impart pushing or pulling force on the central ring to move it from a position proximal to the right side of the frame (frame^R^) toward the left side of the frame (frame^L^) (Fig. 1d). These domains are termed “extended core” (EC), “cinchers” (Ch) and “loops” (L). The corresponding staple strands are termed EC^S^, Ch^S^, L^S^, respectively. The L domain function is illustrated in Fig. 1e, scenario 1. Upon hybridization to L^S^ staples, the L domain extension causes the ring to move away from frame^R^. The Ch domain contains stretches of scaffold DNA that are shorter in length (35 bases) than the underlying EC domain (70 bases) (Supplementary Fig. 3). As shown in Fig. 1e, scenario 2, these domains pull the ring towards frame^L^. Finally, extension of the EC domain by hybridization to EC^S^ staples generates a pushing force on frame^L^, thereby moving it away from frame^R^. This motion results in extension of cincher DNA which pulls on the ring, moving it away from frame^R^ (Supplementary Fig. 3). These design features are illustrated in caDNAno layouts corresponding to each configuration in Supplementary Figs 4–7.

Motion of the ring induced by hybridization of force domains can be challenged by introducing resisting adhesive force(s) at the interface (“active sites”) of the ring and frame^R^ (Fig. 1c). Two types of resistance were tested in this study. The first type is blunt end base stacking (pi-bond interation) at the active sites (Fig. 1f). Previous work has shown that base stacking interactions can result in strong helix-helix adhesion and this interaction has been utilized to create multiunit self-assembling DNA nanostructures^32^,^33^. Previous studies also suggest that GC dinucleotides form the strongest stacking interaction^34^. Therefore, the nanosystem design used in this study employed GC dinucleotide base stacking to promote adhesion at the active sites (Supplementary Fig. 10).

Another kind of resistence is a “lock and key” system, the design of which is based on toehold-mediated strand displacement^25^ (Fig. 1g). Toehold-mediated strand displacement is a dynamic hybridization event wherein a DNA strand invades and displaces another strand from a duplex by binding to a short single-stranded olionucleotide extension called the toehold. In these experiments the active sites were decorated with toehold-bearing duplexes that tether the ring to frame^R^.

### System Characterization and Demonstration

**Figure 2:**
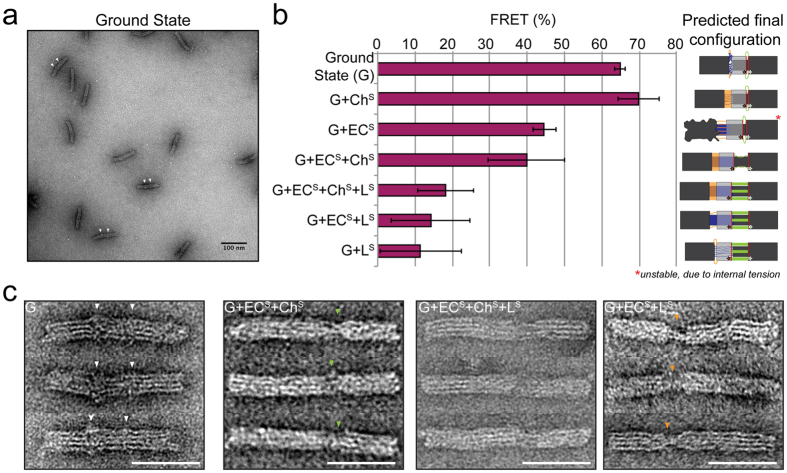
System characterization and demonstration using FRET and Transmission Electron Microscopy (TEM). (**a**) A TEM field showing examples of the ground state of OPTIMuS. White arrows adjacent to some structures indicate single-stranded “clouds” of DNA corresponding to the unhybridized force domains. (**b**) FRET output of OPTIMuS as a function of combination of pre-added (during self-assembly) force domain components. FRET is a reliable indicator of ring/frame^R^ distance and, therefore, a reporter of force-induced ring motion. A schematic of each configuration is shown for clarity. In case of G + EC^S^, pre-adding EC^S^ affects the stable formation of the structure due to internal tension between Ch and EC, thereby destabilizing the formation of the frame (Supplementary Fig. 1). This is one type of internal tension that comprises the foundation of mechanical actuation in the nanosystem. (**c**) Corroborating TEM structures corresponding to key OPTIMuS configurations used in this study (Scale bar = 50 nm). Green and yellow arrows indicate structural “gaps” corresponding to ssDNA L and ssDNA Ch domains respectively.

To assess the scope of influence of each force inducing domain (EC, Ch, L) on the central ring and its effect on the ensemble FRET output, we assembled the nanosystem in the presence of different combinations of domain-specific staples (Fig. 2). OPTIMuS at “ground state” (G) is defined as the configuration that contains no force domains (EC, Ch, and L remain single-stranded). In each sample, staples associated with G (i.e., frame^R^, frame^L^, core and ring) were mixed with active site staples conferring blunt end formation and also a specific combination of force domain staples. After mixing, the nanosystem was assembled following the standard protocol (Methods). The FRET output reflects the position of the ring relative to frame^R^ in each configuration (corroborated by Transmission Electron Microscopy (TEM) analysis; Fig. 2). Since blunt end stacking is strongly distance-dependent^35^, and therefore cannot acquire sufficient force when assembled simultaneously with opposing force domains, the force domains prevail in all cases except Ch^S^ (see following) and induce various degrees of ring movement. Figure 2b shows that Ch^S^ alone has no effect on FRET compared to G. In contrast, L^S^ alone and EC^S^ + L^S^ elicit the same FRET as all three force domains in combination (EC^S^ + Ch^S^ + L^S^). Finally, G + EC^S^ and G + EC^S^ + Ch^S^ have comparable FRET outputs. Thus, these two force domain combinations, while differing in total ΔG appear to reach a common final mechanically limited state of the nanosystem (minimum FRET). Based on these results, we chose three combinations of force domain staples as actuators, EC^S^, EC^S^ + Ch^S^ and EC^S^ + L^S^, to study their effect on the motion of the ring when opposed by two different kinds of resistant forces, base stacking and DNA-DNA hybridization.

### Base stacking vs Force domains

**Figure 3:**
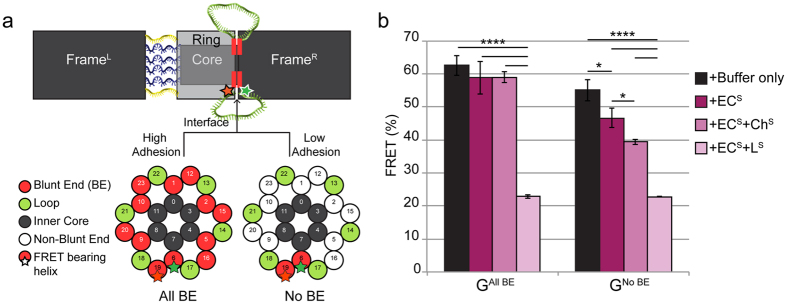
Base stacking adhesive forces versus OPTIMuS actuators. (**a**) The interface makeup between the ring and frame^R^ shown as a cross-section of OPTIMuS. It can be modified with coaxial blunt ends (BE) to create high adhesion or no blunt ends to minimize adhesion. (**b**) FRET output of all blunt ends (All BE) versus no blunt ends (No BE) OPTIMuS upon the addition of different force domains (****Indicates P ≤ 0.0001; *Indicates P ≤ 0.05).

Figure 3 shows the results of experiments in which combinations of force inducing domains were tested for their ability to disrupt base stacking-mediated ring/frame^R^ adhesion. Ground state with blunt ends on all active sites (called G^All BE^) and ground state with no blunt ends on all active sites (called G^No BE^) were initially assembled in the absence of force domains. The samples were divided into equal amounts, filtered to remove excess staples, and then incubated with either buffer alone, EC^S^, EC^S^ + Ch^S^ or EC^S^ + L^S^ (Methods).

In the case of G^All BE^ (all blunt ends stacked) the combination of duplex formation in EC and L domains resulted in rupture of the adhesive bond between the ring and frame^R^. In contrast, EC^S^ + Ch^S^ failed to disrupt the base stacking interaction. We hypothesize that this is the consequence of fully-extended and strained cincher ssDNA being unable to form a sufficient number of hydrogen bonds to initiate an ssDNA to dsDNA transition and thereby create a resultant pulling force (in contrast to ease of duplex formation when cinchers are hybridized during the initial self-assembly process, Fig. 1e Scenario 3; Fig. 2b). Unlike strained cincher domain ssDNA, ssDNA in the loop domain is not stretched but, rather, randomly coiled and, therefore, stereochemically available for hybridization with complementary oligonucleotides. Thus, hybridization to loop domains generates a pushing force on the ring that is sufficient to rupture of the ring/frame^R^ adhesive interaction.

When OPTIMuS was configured to lack base stacking interactions at the ring/frame^R^ interface (Fig. 3b G^No BE^) all combinations of force domains tested were able to induce ring displacement. In particular, duplex formation of EC + Ch was able to disrupt the ring/frame^R^ interface because there was no opposing adhesive force and, therefore, no hyperextension of the ssDNA-cincher domains to preclude cincher duplex formation (Fig. 1e Scenario 2). The results of these experiments suggest that OPTIMuS may be useful for interrogating other types of molecular interactions at the ring/frame^R^ interface.

**Figure 4:**
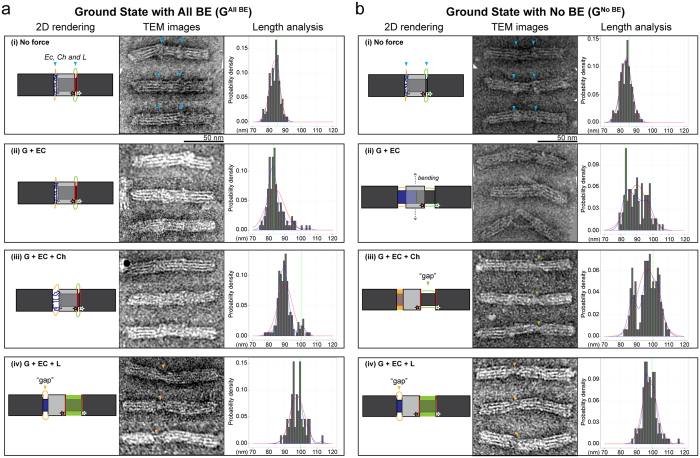
Base Stacking Adhesive Forces versus OPTIMuS actuators: TEM analysis. For each sample, a two-dimensional (2D) rendering was generated using corresponding caDNAno layout, followed by TEM imaging, extraction of three representative images and histrograms showing the density distribution of the overall length of OPTIMuS populations in various configurations. Red curve represents the Normal distribution, blue curve shows the actual distribution and the green dashed line indicates the average length of OPTIMuS in the corresponding pre-added force domain configuration (based on data acquired in Fig. 2). (**a**) TEM images of ground state containing all blunt ends in the presence of different force domains. (**b**) A schematic of the blunt end and non-blunt end interactions between the coaxial helices of ring and frame^R^.

TEM analysis was carried out to corroborate FRET analysis of the various configurations of OPTIMuS. Figure 4 shows that with full bunt end stacking (G^All BE^ ) internal reconfiguration does not take place in the presence of EC^S^ or EC^S^ + Ch^S^ (Fig. 4a(i–iii)). However, the addition of subsequent force domains (EC^S^ + L^S^) induces a change that leads to ring movement (Fig. 4a(iv)). This can be verfied by observing the “gap” inside OPTIMuS which shows the ssDNA cincher domain and helps locate the relative position of the ring (TEM images, Fig. 4a(iv)). The overall length of OPTIMuS is also a good indicator of internal reconfiguration, as can be seen by the dimensional analysis of a population of each kind of sample (right columns, Fig. 4a,b). The mean length of G^All BE^ remains the same upon addition of EC^S^, but shifts to an intermediate length in case of EC^S^ + Ch^S^. The partial hybridization affects the overall length of the nanosystem, but is unable to cause motion in the ring, hence the FRET signal does not alter. This observation supports our hypothesis that there is partial hybridization in the two force domains, EC and Ch, but complete hybridization is stereochemically hindered (i.e., Ch is physically constrained in a stretched configuration) by the blunt end stacking at the active sites.

In contrast to the results above, the configuration lacking blunt end stacking, G^No BE^, undergoes an incremental shift in the ring position as well as length of the nanosystem as a function of force domain hybridization (Fig. 4b). The addition of EC^S^ alone causes OPTIMuS to assume a bent configuration (Fig. 4b(ii)). This can be attributed to a fully-extended ssDNA Ch domain, the tension in which is sufficient to distort the otherwise linear core architecture. This bending serves as evidence that hybridization of the EC^S^ to EC domain is occuring with high efficiency. Duplex formation in the remaining two force domain samples, EC^S^ + Ch^S^, EC^S^ + L^S^, results in full shift in the ring’s position and a corresponding overall increase in the length of the nanosystem. The configuration-specific gaps corresponding to single-stranded Ch and L domains permit unambiguous orientation determination of the molecule and indicate the internal position of the ring (Fig. 4b(iii,iv)).

### Detection of a soluble ssDNA viral genome mimic

**Figure 5:**
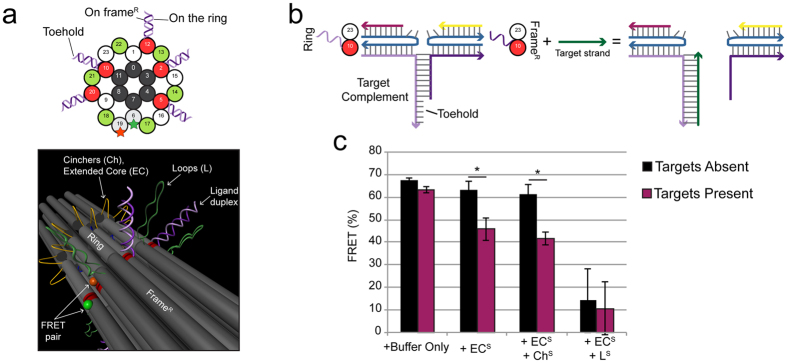
Programming OPTIMuS for nucleic acid detection. (**a**) Cross-section of the interface displaying the placement of the target-associated duplexes. Five sites were remodeled to contain unique duplexes such that the toehold-bearing strand emerged from a staple on the ring and its complement emerged from frame^R^, as shown in the 3D rendering. The duplex contributes to ring-frame^R^ adhesion. The remaining active sites were modified to the no blunt end state, as described in Fig. 3. (**b**) The basic scheme of toehold-mediated DNA strand displacement. A target strand invades the duplex to hybridize with the toehold-bearing strand, which allows the two components to separate from each other. (**c**) FRET readout in different configurations showing that under force induction the ring/frame^R^ interface is disrupted only in the presence of soluble target oligonucleotide (*Indicates P ≤ 0.05).

The earlier suggestion that OPTIMuS might serve as a useful molecular detection platform was tested using a strand displacement mechanism and a viral genome (DNA) mimic. Toehold-bearing duplexes, called ligand domains, were integrated with the ring/frame^R^ interface such that one strand of each duplex extends from the ring and the other from frame^R^. The toehold-containing strand was designed to be complementary to a soluble target oligonucleotide based on five Ebola genome sequence elements (Supplementary Information). The active sites were modified with these ligand domains (Fig. 5a,b). Addition of the target strands disrupted the ligand duplex through toehold-mediated DNA strand displacement^25^, thereby reducing ring/frame^R^ adhesion and permitting free motion in the ring. We compared ring motion in the presence and absence of target molecules in different OPTIMuS configurations. Following the format of experiments that tested blunt end stacking interactions in Figs 3 and 4, we constructed the ground state in the absence of the force domains (G^No BE^) but bearing the ligand duplexes at the active sites (Fig. 5a). The ground state sample was divided, purified via filtration to remove excess staples, and incubated (Methods) with the force domains with or without target strands and FRET was measured. Upon force induction in the absence of target the FRET signal only changed in the presence of the strong disruptive force domain combination EC^S^ + L^S^. However, in the presence of soluble target the FRET signal was significantly reduced when the ring was induced to move using the weaker force-generating domains EC or EC + Ch, thereby illustrating that the OPTIMuS platform has the potential to serve as a molecular detection platform (Fig. 5c).

## Discussion

We describe a self-assembling DNA nanosystem termed OPTIMuS that is capable of interrogating molecular interactions by exerting user-controllable forces to challenge the molecular system of interest. Controlled exertion of force in OPTIMuS is founded on the inherent elasticity of ssDNA (a relatively weak and compliant spring), the shortening and stiffening of double helical DNA (a relatively strong and stiff spring), the high specificity of DNA base pairing, and the adhesive force exhibited by base stacking. The availability of a plurality of control elements should allow OPTIMuS to be tuned to interrogate interactions of a range of strengths. In the present study we explore a soluble target strand displacement mechanism of detection and the interactive force present in base-stacked DNA duplexes.

A self-assembling DNA-based system that is capable of interrogating and, potentially, measuring inter- and intramolecular forces/energies is compelling for several reasons. It is extremely economical in comparison to macroscopic instrumentation that is used for molecular force measurements (e.g., atomic force microscopy (AFM) and optical trapping). Moreover, in contrast to those systems OPTIMuS has the potential to perform thermodynamically reversible force induction, which would overcome the limitations of time-varying external forces obtained by AFM and optical tweezers^36^. The strength of each force domain may be tuned at the single base pair level to create a highly nuanced spectrum of test energies. Finally, this system may lend itself to statistically robust soluble molecular population-based as well as chip-based single molecule or smaller population analyses.

As a sensor, OPTIMuS is readily reconfigurable and capable of multiplexing, a potential advantage over molecular beacons^37^. Unlike one-dimensional DNA-based sensors, OPTIMuS allows the ability to use bulk FRET, corroborated by TEM output to detect molecular states. DNA is amenable to a wide range of chemical modifications making it relatively simple to incorporate a variety of molecular species into the system for study. Versions of self-assembling systems like OPTIMuS can be multiplexed to create (AND/OR) logic gates and iterative biosensors for high confidence molecular detection. Moreover, the ability to precisely arrange gold nanoparticles on OPTIMuS^38^,^39^ suggests a pathway to enhanced sensitivity by methods such as surface-enhanced Raman spectroscopy (SERS) for use in field deployable diagnostics^40^. Finally, DNA nanosystems are inherently biocompatible and may be further embellished to create novel bionanodevices that have the potential to interact with natural biological systems *in vivo*.

## Methods

### Nucleic acids

All oligonucleotide staple strands were purchased from Integrated DNA Technologies (IDT, Coralville, IA), supplied in RNase-free water at 100 μM concentration in individual wells. M13mp18 single-stranded scaffold DNA was purchased from Bayou Biolabs (Matairie, LA) and was supplied at a concentration of 1 μg/μL in Tris-Acetate EDTA buffer. Experiments were carried out without additional purification steps.

### Chemical and supplies

All other chemicals (Tris-Acetate EDTA, Magnesium Acetate Tetrahydrate, and water) and supplies were purchased from Fisher Scientific.

### Assembly of OPTIMuS

The annealing protocol was adopted from Stein *et al.*^41^. The requisite staple strands (including the fluorescently-labeled staples), each at a final concentration of 50 nM, were mixed with m13mp18 scaffold strand at a final concentration of 10 nM in 1× reaction buffer (comprised of 40 mM Tris-Acetate, 1 mM EDTA (pH 8.3) and 18 mM Mg^2+^) and brought to a final volume of 500 μL. The desired structures were assembled using the following thermal annealing program:

80 °C – 5 min

80 °C to 60 °C – 80 min

60 °C to 25 °C – 1200 min

25 °C to 4 °C – 10 min

4 °C – storage until further experiments.

Care was taken to maintain all samples in the dark by covering the PCR plate as well as the laboratory tube rack with aluminum foil.

### Centrifugal filtration

Removal of excess staples, particularly those with fluorescent labels, was critical for optimal results and quantification. Excess staples were removed using Amicon Ultra-0.5 mL Centrifugal Filters (50,000 molecular weight cutoff (MWCO)). 500 μL of the reaction mix was poured into a filter column and centrifuged at 14,000 g for 5 min. The eluate collected in the collection tube was discarded and the filter column was placed back into the collection tube. Centrifugation step was repeated 4 times on the same filter column by adding 450 μL of 1× reaction buffer to the filter column before each step. After completing the centrifugation, the retentate was recovered by inverting the column in a fresh tube and performing centrifugation (at 1000 g) for 3 min.

### Agarose gel electrophoresis

The efficiency of assembly was evaluated by electrophoresis using a 1.5% agarose gel. Electrophoresis was carried out on ice at 72 Volts for 4 hours. Gels were stained with 1× SYBR Green and illuminated under UV (302 nm) using a Benchtop 2UV^TM^ Transilluminator (UV Products).

### Post-assembly sample treatment with different combinations of force strands and target strands

In experiments in which a preconfigured OPTIMuS sample was treated with force domain strands or target strands (Figs 3b and 5c), filtered samples were mixed with 100 nM of each desired staple, such as extended core, cinchers and loops. The buffer conditions of the force domain staples were consistent with the ground state sample and the cation concentration of the resultant samples was maintained at 18 mM Mg^2+^. Samples were incubated using the following thermal annealing protocol:

40 °C – 60 min

40 °C to 25 °C – 600 min

25 °C to 4 °C – 10 min

4 °C – storage until further experiments.

### Ensemble fluorescence resonance energy transfer (FRET)

In order to minimize background fluorescence, only filtered samples were used for FRET experiments. A custom-designed well was created in lab using a microscope slide and a coverslip to carry out fluorescence microscopy through a hyperspectral microscope (Nikon Eclipse TE2000-E) and EXFO X-Cite 120 PC Fluorescence illumination system. Exposure time was set at 50 ms and a 20X objective was used to image using an HQ Wide Green Filter (Excitation Filter: 545/30 nm, Dichromatic Mirror: 570 nm, Barrier Filter: 610/75 nm). Data acquired with the hyperspectral microscope for each sample was a two-dimensional array of Wavelength (nm) and Intensity (arbitrary unit). Intensities were normalized by the total intensity (i) received per sample before calculating FRET. FRET was calculated based on equation (1):


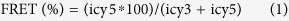


where, icy3 – fluorescence intensity at cy3 emission (574 nm); icy5 – fluorescence intensity at cy5 emission (669 nm).

### Transmission Electron Microscopy (TEM)

Sample preparation for TEM imaging was based on the protocol described by Castro *et al.*^42^. Briefly, 12 μL of the sample solutions (~10 nM concentration) were placed on glow-discharged carbon-coated 400 mesh copper TEM grids. After 2 min, the samples were wicked off of the grid with filter paper and immediately replaced with 12 μL of freshly prepared uranyl formate negative staining solution. After 30 sec, the stain was removed and the grids were allowed to air dry. Images were acquired at 25,000x using a JEOL 1230 TEM (Peabody, MA) equipped with a Gatan Inc. 2k × 2k Ultrascan camera (Pleasanton, CA).

### Image processing

TEM imaging generated .dm3 files, which were fed into the boxer.py program of EMAN2 to pick individual particles (or nanostructures) and create a stack. Then we performed dimensional analysis (length measurement) using the line tool in ImageJ or Fiji. Scale of the images was set according to the information stored in the .dm3 files. Histograms were generated with the help of a simple R code.

## Additional Information

**How to cite this article**: Mathur, D. and Henderson, E. R. Programmable DNA Nanosystem for Molecular Interrogation. *Sci. Rep.* **6**, 27413; doi: 10.1038/srep27413 (2016).

## Supplementary Material

Supplementary Information
