# Supplementary material for: Programmable DNA Nanosystem for Molecular Interrogation
Source: Sci Rep. 2016 Jun 7;6:27413. doi: 10.1038/srep27413 (PMC4895238; doi:10.1038/srep27413)
Supplement: Supplementary Information [file srep27413-s1.pdf]

Supplementary Information for

Programmable DNA Nanosystem for Molecular Interrogation

Authors: Divita Mathur[1,2,*], Eric R Henderson[1,2,*]

Affiliations:

[1]Department of Genetics, Development and Cell Biology, Iowa State University, Ames, IA 50011, USA.

[2]Bioinformatics and Computational Program, Iowa State University, Ames, IA 50011, USA.

[*]Correspondence to: divita@iastate.edu, telomere@iastate.edu

Table of Contents

Supplementary Note S1: Design Principles in OPTIMuS 3

S1.1 G+EC pre-added condition 3

S1.2 Design of OPTIMuS 4

S1.3 Agarose gel electrophoresis analysis 9

S1.4 Placement of fluorophores for FRET 9

S1.5 Design of the blunt end interface 9

S1.6 The ligand domains 10

Supplementary Note S2: Staple and target strands 11

S2.1 DNA sequences 11

Supplementary Note S3: References 16

Supplementary Note S1: Design Principles in OPTIMuS

S1.1. G+EC pre-added condition

Due to internal tension between the shorter Ch domain and the EC domain, initial assembly of OPTIMuS in the presence of the EC staples can destablize the structure. As the EC staples bind to their respective scaffold segments, they extend to their full duplex length, which induces hyperextension in the Ch domain. This hyperextension prematurely causes a pulling force on the frame-l, thereby preventing the complete assembly of frame^{L}. Supp. Fig. 1 shows three example TEM images of OPTIMuS (G+EC^{S}) wherein frame^{L} is irregular in shape compared to the right side (frame^{R}).

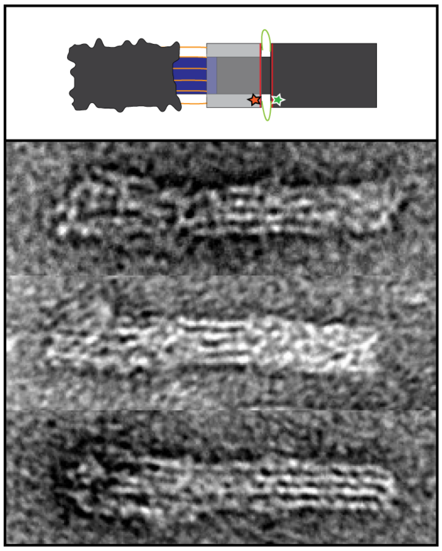

Supplementary Figure 1. Assembly of G in the presence of EC^{S} (Preadded condition).

S1.2. Design of OPTIMuS

OPTIMuS is designed using one long m13mp18-based scaffold strand that follows a raster pattern along a 24-helix bundle cylinder to create frame elements (frame^L, frame^R, inner core and ring) as well as the force generating domains (extended core , cinchers and loops) (Figure 1b). The structure is designed in the honeycomb lattice of caDNAno, a design tool to create DNA origami architectures, using standard crossover rules. The scaffold raster layout is in a "radial" fashion, i.e., the scaffold directly links each inner core helices to the nearest outer layer helix (Supp. Fig. 2). Staples corresponding to the ring do not attach to the inner core, which enables free motion of the ring. To minimize ring rotation about the core axis, the ring/frame^R interface has a "staggared" design, analogous to the edge of a key corresponding to a lock (Supp Fig. 4), but for simplicity, the renderings in the main text show a linear ring/frame^R interface. The ends of the nanostructure contain single-stranded scaffold domains to inhibit unfavorable intermolecular base stacking-based polymerization. The 18 pairs of coaxial helices at the arbitrarily designated right side ring-frame interface (ring/frame^R) are a homogeneous arrangement of 6 loop domains, 2 fluorophore-bearing helices and 10 active site domains (Fig. 1c).

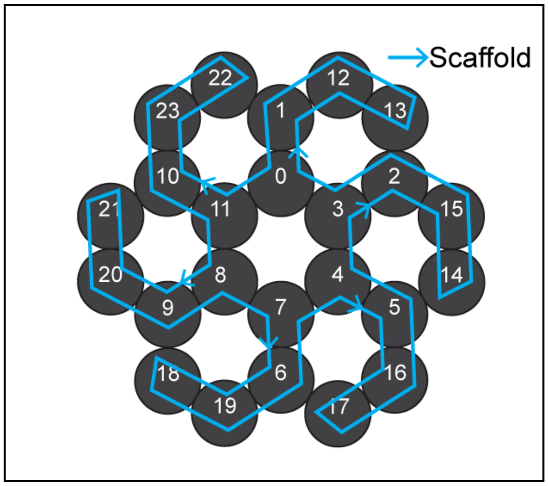

Supplementary Figure 2. Scaffold raster layout. The raster follows a "radial" path, where each inner core crosses over to the outer helices in the 18 hb before crossing back to the inner core. This enables homogeniety and symmetry in design.

We incorporated the new scaffold layout strategy proposed by Ke et. al.[1] to improve assembly and yield. The contact order for scaffold raster ("average sequence distance between scaffold segments that are brought into close proximity by staple strand interaction") was chosen to be close to 42 bp without adversely affecting the functionality of the nanosystem (Supplementary Figs. 4-7).

Force domain design. All force domains (EC, Ch, L) are single-stranded domains of the scaffold that bind to their complementary force domain staples (EC^S, Ch^S, L^S) and elicit a physical change in the relative position of the central ring within OPTIMuS. Therefore, the force domains are covalently attached (through DNA phosphodiester bond) to the frame elements – frame^R, frame^L and ring. This imparts highest possible robustness and stability to the force domains.

The left side ssDNA domains plays a dual role of establishing a rigid anchor for the ring as well as inducing a pulling force on the ring, which directly couples this part of the system to the FRET reporter system. The figure below (Supplementary Fig. 3) illustrates the relative state

change in the ring by ssDNA-dsDNA transitions in EC and Ch. In this figure, we show that $Frame^L$ is bound to the remaining body of OPTIMuS via the force domains EC and Ch. In the ground state, the $Frame^L$ is positioned relatively proximal to the main body due to the anisotropic elasticity of the ssDNA (i.e., a weak spring in an averaged ground state of compression-extension). This condition holds the ring in position near $Frame^R$ as can be seen by TEM data as well as high FRET output (Fig. 2). Upon the addition of EC^S and the subsequent ssDNA to dsDNA transition in the EC domain, the EC domain transitions from a weak spring to a rigid rod composed of several double helices. The rigidity of the rod results in pushing of the $Frame^L$ domain away from $Frame^R$. Critically, $Frame^L$ is tethered to the central ring by the cincher (Ch) domains, which are stretched/strained by extension of the EC domain. Thus, the cinchers exert a pulling force on the ring and move it away from the ring/$Frame^R$ interface, resulting in FRET output diminution. A hybridization-induced ssDNA to dsDNA transition in the cincher domains imparts an additional pulling force on the ring and stabilizes it in the intermediate $Frame^R$ distal position (intermediate FRET).

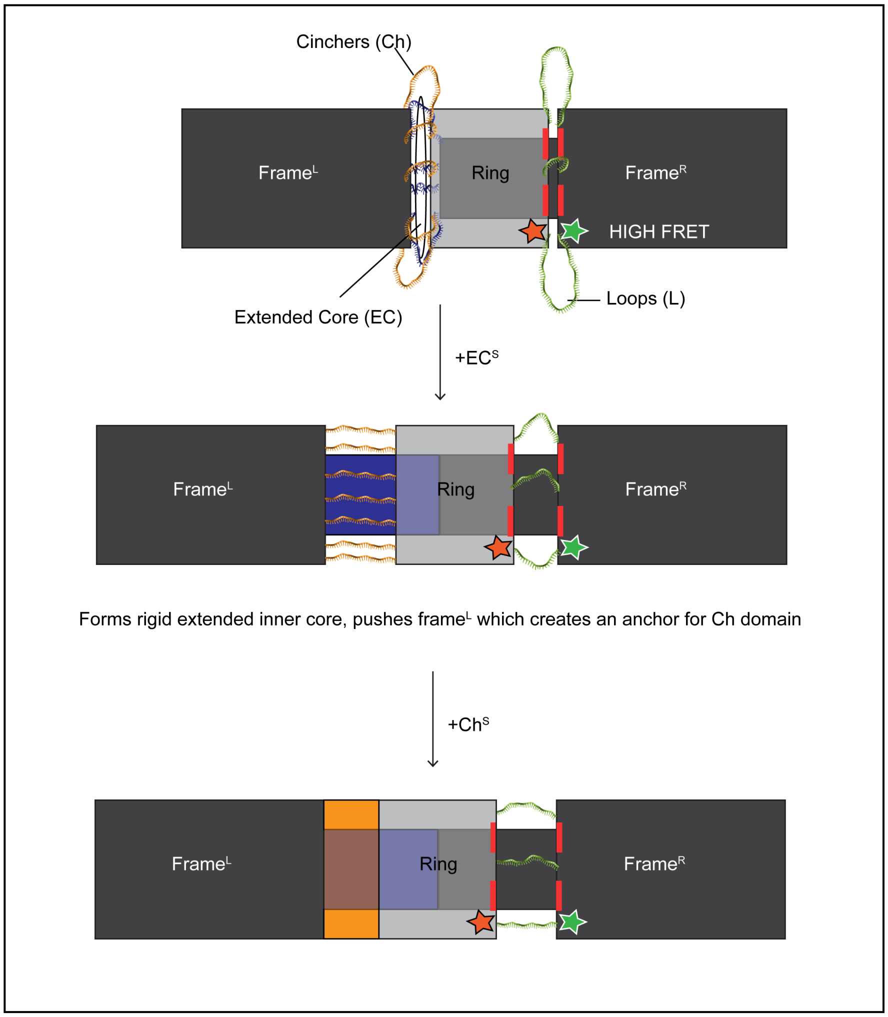

Supplementary Figure 3. Role of EC domain in ring movement. This figure illustrates the programmed motion in the ring induced by the duplex formation in EC domain, followed by the duplex formation in Ch domain. The EC domain, upon hybridizing with complementary staples creates a rigid tether between $frame^L$ and the inner core. This process leads to the extension of Ch domains, which pull the ring towards $frame^L$.

Different conformations of OPTIMuS arise from the binding of staples associated with different domains on the scaffold. Below are caDNAno renderings of OPTIMuS in different conformations.

The ground state (Supp. Fig. 4) is the initial conformation of OPTIMuS in the absence of any staples associated with the force domains (no EC, Ch, Loops). The scaffold domains corresponding to EC, Ch and L are single stranded in solution. The ring-frame^{R} interface, which forms the active site, can be modified to contain blunt-end stacking, no blunt end stacking or toehold-bearing duplexes.

Supporting figures 5, 6 and 7 show OPTIMuS with force domains bound to their predefined scaffold domains as well as active sites modified to contain GC blunt ends.

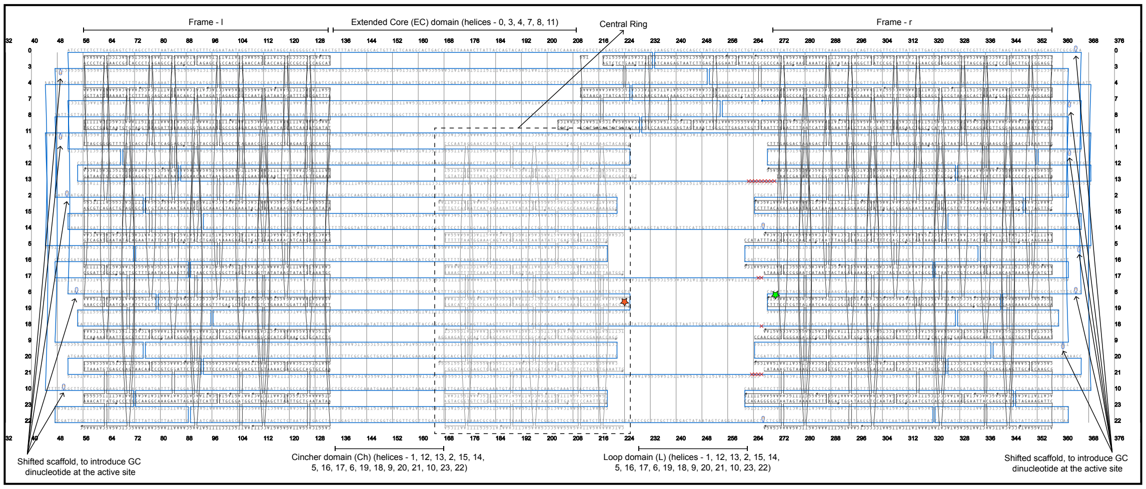

Supplementary Figure 4. caDNAno design of OPTIMuS: Ground state. This is the ground state (G) without any hybridized force domains. The nanosystem is a 24 helix bundle (hb) where each helix contains approximately 316 bp. Cy3 and Cy5 molecules are shown in green and red star respectively.

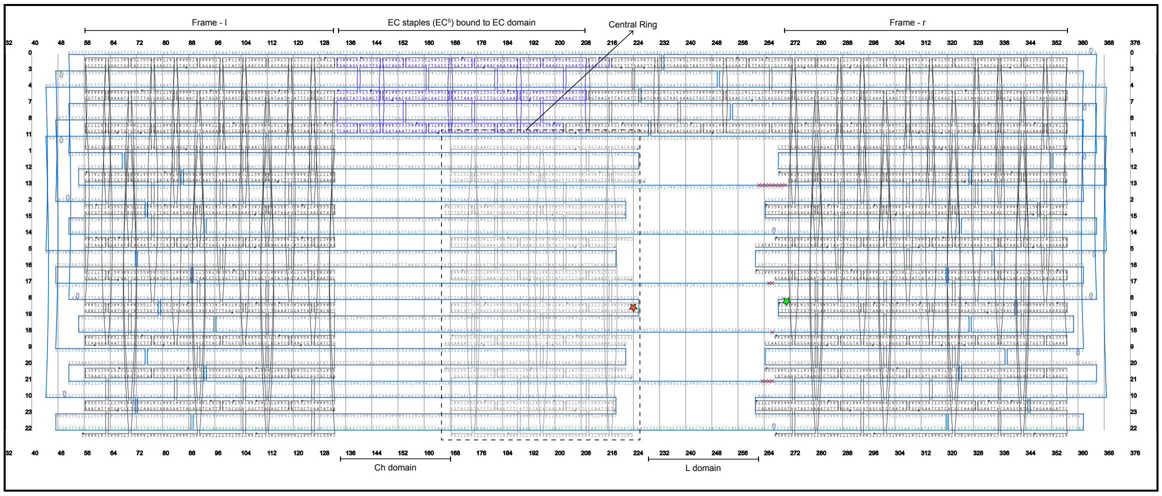

Supplementary Figure 5. caDNAno design of OPTIMuS: G + EC^{S}. The staples corresponding to the Extended Core (EC^{S}) domain are shaded in violet, which bind to the extension of the inner core (EC).

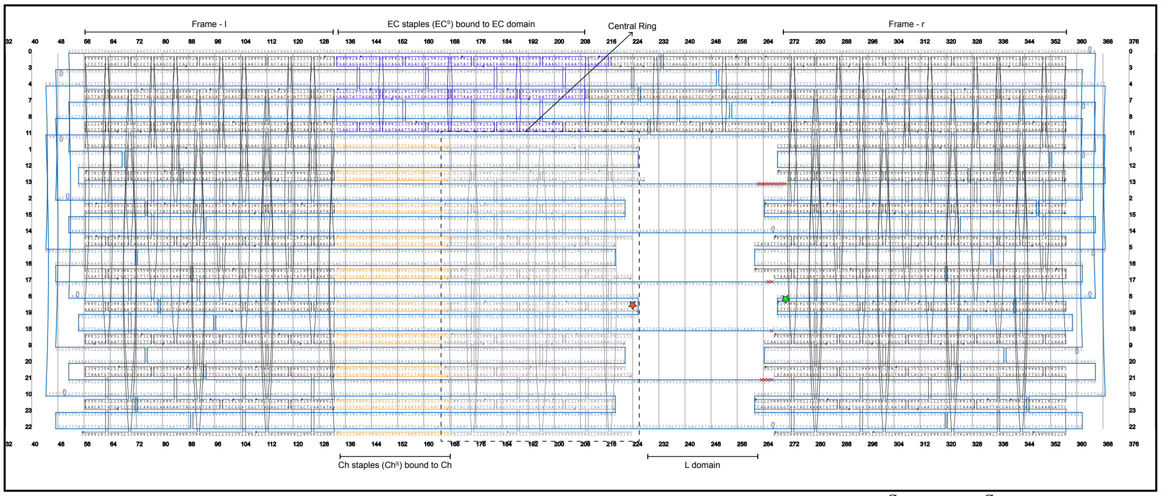

Supplementary Figure 6. caDNAno design of OPTIMuS: G + EC^{S} + Ch^{S}. The staples corresponding to the Extended Core (EC^{S}) domain are shaded in violet, which bind to the extension of the inner core (EC) and orange Cincher staples (Ch^{S}) bind to the Cincher domain (Ch). Concequently, the ring's relative position should change.

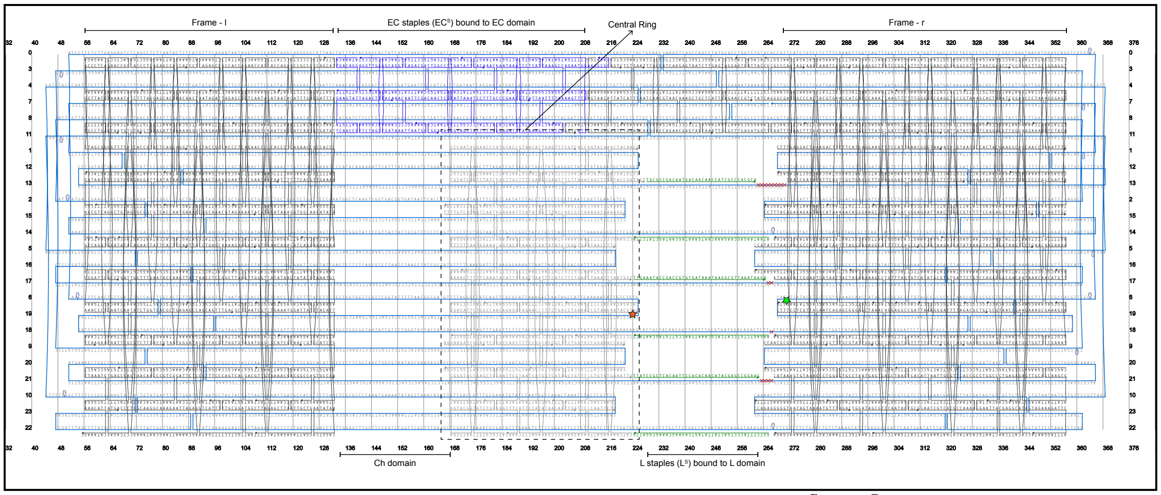

Supplementary Figure 7. caDNAno design of OPTIMuS: G + EC^S + L^S. The ground state. The staples corresponding to the Extended Core (EC^S) domain are shaded in violet, which bind to the extension of the inner core (EC) and dark green Loop staples (L^S) bind to the Loop domain (L). Concequently, the ring's relative position should change.

S1.3. Agarose gel electrophoresis analysis

Supplementary Fig. 8 depicts the agarose gel electrophoresis analysis of six primary configurations of OPTIMuS. OPTIMuS formed in the presence of force domains (extended core, cinchers and loops) migrated further than their counterparts due to additional stability imparted by the hybridized force domains.

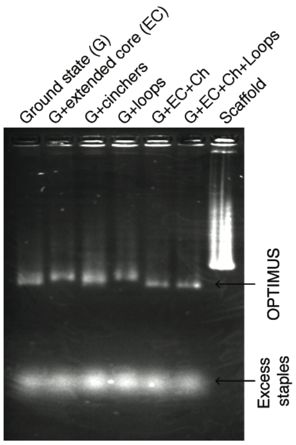

Supplementary Figure 8. Agarose gel electrophoresis analysis of OPTIMuS.

S1.4. Placement of fluorophores for FRET

Designing of fluorophore-labeled staples was inspired by the work done by Stein et. al.[2] A staple strand on the ring was modified with a cyanine 5 (cy5) molecule whereas a staple on frame^{R} was modified with a cyanine 3 (cy3) molecule. Care was taken to ensure optimum stereochemical alignment of cy3 and cy5 molecules in accordance with the helicity of DNA. Supplementary Fig. 9 shows the predicted position of cy3 and cy5 along the ring/frame^{R} interface.

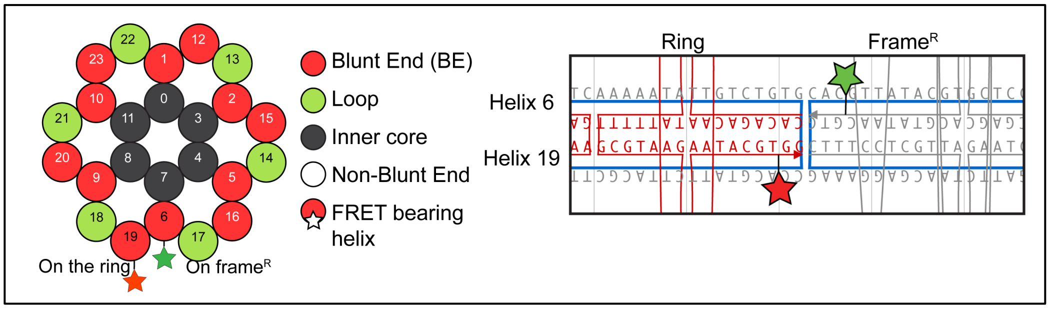

Supplementary Figure 9. Fluorophore placement on OPTIMuS. Cy3 (green stae) was positioned on frame^{R} and cy5 (red star) on the ring.

S1.5. Design of the blunt end interface

The energetics of blunt end adhesion is correlated to the nature of participating bases. Previous literature demonstrates that GC/GC at the blunt ends has strongest attractive force (-2.17 kcal mol^{-1}). Therefore, to create an energetically homogeneous and the strongest possible adhesive interface, GC dinucleotides were desirable at the interacting end of the ring and frame^{R}. Woo and Rothemund[3] in their online supporting information have shared a modified version of

caDNAno software, called caDNAnoSQ_SW that assists in monitoring the position of all GCs (or any other desired dinucleotide) along the scaffold while designing a nanostructure. With the help of caDNAnoSQ_SW the scaffold sequence was inserted into the structure design such that the crossovers along the ring/frame^R interface were populated only with GC dinucleotide by circularly permuting the scaffold until a GC pair was present at each active site (Supplementary Fig. 4-7, 10). Extra scaffold remaining from the permutation was positioned at the ends of helices to assist in preventing unwanted inter-OPTIMuS stacking interaction.

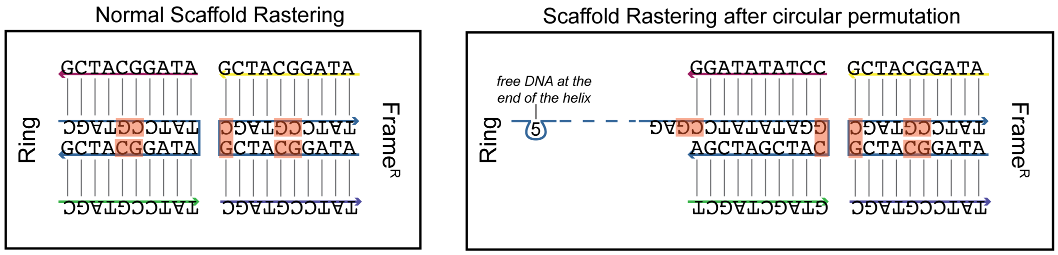

Supplementary Figure 10. GC/GC blunt end active site. Using caDNAnoSQ_SW, GC dinucleotides along the scaffold were highlighted and the scaffold was shifted by introducing loops at the ends of the helices (that contains some free DNA to prevent unfavorable interactions between nanostructures.

S1.6. The ligand domains

To demonstrate the application of OPTIMuS as a diagnostic tool, we designed five nucleic acid-based ligand domains at the ring/frame^R interface that are specific to unique target sequences. The ligand domains were introduced into OPTIMuS by appending the ligand duplex sequences to the ends of two staples at the ring/frame^R interface such that one extended from the ring and the other from frame^R. Each target strand is a subsequence from the Ebola genome (GenBank Accession Number: KM233090.1). The NCBI Primer BLAST primer-designing tool was employed to identify unique 20 bp target sequences from the genome. After identifying target strands, the ligand domain duplex was designed such that one strand was completely complementary to the target while the other strand was identical in sequence to the target strand but was 5 bases shorter in length. This created a toehold in the duplex to initiate DNA strand displacement by the soluble target strand. The staples strands modified with the ligand domains and the target strand sequences are included in Supplementary Table 1 below.

Supplementary Note S2: Staple and target strands

S2.1. DNA sequences

Name	Ring or Frame	Sequence	Length	Color in Supp fig. 1
$Frame^L$ 1		ATGATGATATCTGAAAAGTGCCAA	24	#333333
$Frame^L$ 2		CCTATGAAAGAAAAGGGCATCCAAGATTCTCCGTTTCCCAGTCACAGTA	49	#333333
$Frame^L$ 3		AAACAGAATTATTCATTGCACCGTGTCACCAATGAAACATTAGCACCAC	49	#333333
$Frame^L$ 4		ATACCTGAGCACTACCTTTCAAAATCGCGCAGAGGCGTAACAGTAGCCT	49	#333333
$Frame^L$ 5		ATGAAACAAATCAAAATCATAGGTGTTGGGTGCGG	35	#333333
$Frame^L$ 6		TTTGGCCGGATATTATTCACCCTCCAGCGGAGTGAGAATAGAATAAGGT	49	#333333
$Frame^L$ 7		CAAGCATCTGGGATGTGCTGCAAGACGGCCAGGTG	35	#333333
$Frame^L$ 8		TGTATACTTTTGAGCGAGTAACAAATGGCCTTCCT	35	#333333
$Frame^L$ 9		GTATTGTTAATCTTTAGTTGGCAATGCTTTGAATACCAAGTTATTTGCA	49	#333333
$Frame^L$ 10		TTAAGCAAAATTAAGCAATAAAGCTCACCGTAGGCTGACACCCTCACGT	49	#333333
$Frame^L$ 11		CCGGAATAGGAGAGGGTAATTTTCATCGGCAGAACCGCGACTCCTGTAA	49	#333333
$Frame^L$ 12		TTTCAGCAAAACATAAAAAGACTCCTTATTAGATG	35	#333333
$Frame^L$ 13		CGGGATAAGTGCCGTCGTGTGCTCAGTACCA	31	#333333
$Frame^L$ 14		CGGTGATATAAGAGGAACAACTAACATTACCCAGCAAACGTAGAAAATA	49	#333333
$Frame^L$ 15		CACAGGAATTGCGAATAACAGTTTAGAACCGCTTT	35	#333333
$Frame^L$ 16		AGCTTTCACGTTGAAAAGATTTTGGCCACCCCAGGTCAAATATAA	45	#333333
$Frame^L$ 17		CATATCACCAGAACCACCGGAACCGACAGTCTACT	35	#333333
$Frame^L$ 18		CAAAGCGTCAGACTGTAGCGCGACAATCAGTAGCG	35	#333333
$Frame^L$ 19		ACAGAATATAACAGAAATAAAATAATCAGCTTAATTCGCGTCTTGATCA	49	#333333
$Frame^L$ 20		ATTGGTGGCAACATATATTGGGAACCAGCATGTTA	35	#333333
$Frame^L$ 21		GAACTGGAAAACAAGGATTATGGATTTATAAG	32	#333333
$Frame^L$ 22		TTATCAAAATAACAACTTTCGCATTGGGATAGGAA	35	#333333
$Frame^L$ 23		ATGGAAGGGTGTCAATAAACGTTAAGATGGGAGTT	35	#333333
$Frame^L$ 24		GAGAAAAGAACGCAGTATGTTATCGATAGCATCAA	35	#333333
$Frame^L$ 25		CAATAGTTAAATGCAGAAGATTTACATTATTGCAACAGGAAATAAC	46	#333333
$Frame^L$ 26		CGGATTCCCTTTTAGTCAGATGAAT	25	#333333
$Frame^L$ 27		GATATCAACACAATCAATGCTGAACCTCAAATCAA	35	#333333
$Frame^L$ 28		ACAGTGCCAGGAGCACTTATTTGCAGAGCCAAGGC	35	#333333
$Frame^L$ 29		TCACAATCGTATGGAAATACCTACTTGGTGTATAT	35	#333333
$Frame^L$ 30		GAGTATATAACTATATGGAATTTACATCAAGCATG	35	#333333
$Frame^L$ 31		AAACCCTGTTGAAAGGTTATCTAAAGAAACCCTCATTTT	39	#333333
$Frame^L$ 32		GCTGTATTAATAGAGCCTAGAACCGCCACCAGTAG	35	#333333
$Frame^L$ 33		CCTTATCTGGTCACGCTGAGAGCCAGCAGTTTGAC	35	#333333
$Frame^L$ 34		CTCCTGAAATGGATTATAAAACAGCATTTGAACTTCTGGCCGCCGTTAG	49	#333333
$Frame^L$ 35		AAACATTTTTTGCCTGACAAGAGATTAGCGGGGTTTTACTCAGAGATGA	49	#333333
$Frame^L$ 36		TAATAAATTTAAGATTCTATTAAGACTCAGGTAGC	35	#333333
$Frame^L$ 37		CGTATATTTACATCAATCCCCCTGCAGAACCCTAAACAACTTTCAATAA	49	#333333
$Frame^L$ 38		GTAGCCAGCTTTAAATGTGCGGGAAAACATTCATAAAGC	39	#333333
$Frame^L$ 39		CAAACGGCGGCCCGTCGTAAATCATACAGTAGTAG	35	#333333
$Frame^L$ 40		GGGTAACGCCAGGGTTGGGTCACGATCAAATGAAAAATCTAAAGCACCG	49	#333333
$Frame^L$ 41		TAATGAGAAAGTTAAAAAATAGATCACCGCCAACCTCCGGCTTAGCTGA	49	#333333
$Frame^L$ 42		GTATTGCGGATGCTCCTTTTGATAGTACCGCCTGAAACCAGAGCCTACC	49	#333333
$Frame^L$ 43		AATGACGTTGTAAAACGGCGATTACGCATCGAACG	35	#333333
$Frame^L$ 44		GCAATTGCTGGGATTAGAGAGTACCCACCCTCCTATTTCACCAGAAATA	49	#333333

Frame^{L} 45	CCCTGGCAAGGCAAAGAATTAGAGGTCATTTGCAT	35	#333333
Frame^{L} 46	AATTGGCTTAGAGCTTATCAATTCAAATCACAATTGTAGATAATAAGGT	49	#333333
Cincher1	TCAGAGCCACCACCCTCATTTTCAGGGATAGCAAG	35	#57bb00
Cincher2	CTTTCCAGACGTTAGTAAATGAATTTTCTGTATGG	35	#57bb00
Cincher3	TCTCCAAAAAAAAGGCTCCAAAAGGAGCCTTTAAT	35	#57bb00
Cincher4	AGGTGAATTATCACCGTCACCGACTTGAGCCAT	33	#57bb00
Cincher5	AAAGAAACGCAAAGACACCACGGAATAAGTTTA	33	#57bb00
Cincher6	ACCGAGGAAACGCAATAATAACGGAATACCCAAAA	35	#57bb00
Cincher7	AATTAATTACATTTAACAATTTCATTTGAATTACC	35	#57bb00
Cincher8	ATAGCGATAGCTTAGATTAAGACGCTGAGAAGAGT	35	#57bb00
Cincher9	TGATGCAAATCCAATCGCAAGACAAAGAACGCGAG	35	#57bb00
Cincher10	ACATCGCCATTAAAAATACCGAACGAACCACCAGC	35	#57bb00
Cincher11	GGCAGATTCACCAGTCACACGACCAGTAATAAAAG	35	#57bb00
Cincher12	TGCTGGTAATATCCAGAACAATATTACCGCCAGCC	35	#57bb00
Cincher13	CCAGTTTGAGGGGACGACGACAGTATCGGCCTC	33	#57bb00
Cincher14	GCCTCTTCGCTATTACGCCAGCTGGCGAAAGGG	33	#57bb00
Cincher15	GCTTGCATGCCTGCAGGTCGACTCTAGAGGATCCC	35	#57bb00
Cincher16	AACCTGTTTAGCTATATTTTCATTTGGGGCGCGAG	35	#57bb00
Cincher17	TGCTGTAGCTCAACATGTTTTAAATATGCAACTAA	35	#57bb00
Cincher18	GCTTCAAAGCGAACCAGACCGGAAGCAAACTCCAA	35	#57bb00
Ring1	ACCGACAGCCCTCATAGGTGAATTGGAGGGAGTTT	35	#cc0000
Ring2	CGTGTAACACAGACTTCATTCCATATAACAGGTTT	35	#cc0000
Ring3	ACAAGTACAAAAGATTACCAATTCTG	26	#cc0000
Ring4	CCTGTAGCAATACCGATAGTA	21	#cc0000
Ring5	TGTATCGTTGTCGTCCCAATAAATTCGAAGTACGGTGTCTGGCGCA	46	#cc0000
Ring6	ATTAATCAATGTTACCAGAAGGAATTTTTTAATGGAAAGAATCCTTGAA	49	#cc0000
Ring7	CGACATTCAACCGATTGAGTCTTAAACAGCTTGTTCC	37	#cc0000
Ring8	TTTTGTCACATTCATTAA	18	#cc0000
Ring9	ACCGTAAGCAGATAGCCTAAATCAATATATGCTATTAATTAATTTTAGT	49	#cc0000
Ring10	AAAAGAAAATTCATATGAGGTAAATATTGACAGCTTGCTTTCGAGTTAG	49	#cc0000
Ring11	ACCGAAGCCCTTTTTATAACCTTG	24	#cc0000
Ring12	TGAAAGAAAAAGCGCCAAAGACAAAAGGG	29	#cc0000
Ring13	AACAAAACTTCCCTAAAGGACATTTCGGCCT	31	#cc0000
Ring14	CTTCTGTAACTAAATTTATTTTTGCGTAAGTTAGTAATAAC	41	#cc0000
Ring15	TAACTTTAATTAGAACCTGAGTAGAAGAACTAGCCAGCTTTCCGGCGCA	49	#cc0000
Ring16	TAGTTTCAAATATATTTTCCCTTACAGTACAGAAC	35	#cc0000
Ring17	TATTAGTTTTCATCTTCTGACATCGTCGTGAG	32	#cc0000
Ring18	CACAGACAATAATGGTA	17	#cc0000
Ring19	ATCTCTGGTGCCGGAAACCAGG	22	#cc0000
Ring20	TCTTTGAAATACGT**/iCy5/**GG	16	#cc0000
Ring21	AGGAAGATCTCGGTGCGG	18	#cc0000
Ring22	TCCCAAACTACTGGCCAACAGAGAGCGCGAACTGA	35	#cc0000
Ring23	GCTACTTGCCCTTCTGACCTGAAAGAATGGC	31	#cc0000
Ring24	ACTGTTGGGAGAATTCGTACATTTAAGTTTCAAAT	35	#cc0000
Ring25	CAAAGCGCCATTCGCCATTGCTGTTTAGATTTATTGA	37	#cc0000
Ring26	AATGGTCAATCGGGTACCGAGCTCAGGGCGAGCAC	35	#cc0000
Ring27	GACCATTAGATAATCATGGTCATACAGGCTGCACC	35	#cc0000

Ring28	CGAACGAGTCCTGTGTGAAATTGG	24	#cc0000
Ring29	TTCAGAGGAAGCCCGAATGAGTTTCGTC	28	#cc0000
Ring30	ATCGCGTTTTGGAACCCATGTACCAACGATCTAAAGTTGTTTATCGGAA	49	#cc0000
Ring31	GCATCAAAACTACAACG	17	#cc0000
EC1	ATAAACATGACAGGATTGTTT	21	#888888
EC2	CGGGGTAGCTATTTTTGATGTACCTCCTTTGCCCGAACCATC	42	#888888
EC3	AACTTCCTGATTATCAGTATTAAACCGGTTGATAATCACGGA	42	#888888
EC4	CTGAGGTTGAGGCAGGTGTAACAGTGATAAATTAATGCGAAA	42	#888888
EC5	ATAAAATAAATCCTCATGGAGTGTTACAAAGGCTATCATAAA	42	#888888
EC6	AACCCCAAAAACAGGAATTCTAGCTGCCCGT	31	#888888
EC7	TTAAGCATGTCAATCATAGAGATCACTGGTAATAAGTTCACA	42	#888888
EC8	AGCAATTCGACAACTCGATGATGGTTGGCCTTGATATTTTAA	42	#888888
EC9	ACTATTTTAAAAGTTTGCACCAGAAGAATGGAAAGCGCTACA	42	#888888
EC10	TCAACCGGATTGTAGAAGTATTAGACTTAATC	32	#888888
EC11	GAGGGTCAGTGCCTTGACAGACGACAATTCATCAATATTACA	42	#888888
EC12	ATTCTTTTGATGATACATAAAGCCAGGAGCGGAATTATGTTA	42	#888888
Core1	TGGGCCTGAGAGTCTGGAGCAAACTCCAGTAAGCGTCAAGTC	42	#888888
Core2	GCGGGCTTGAGATGGTTCAAAGGGCGAAATGAATAAGGTAAA	42	#888888
Core3	ACAGAATTACCGCGTTTTTATTTTCAAGAACCGTC	35	#888888
Core4	TCTCGGAACAAAGAAACAGTAACAATGAACGGTAATCGGGTC	42	#888888
Core5	TTACCATCAAGAGTAATCTTGAGTAGGAATCCCAG	35	#888888
Core6	TCGTTATCATTTAATCAACGTAACTGACGAGAAACAAGAGAA	42	#888888
Core7	CCCAAAGCTGCTCATTCTCCAAGAACCGGATATTCATTACCCATGGAAT	49	#888888
Core8	TTGCATAGGCTGGCTGACCTTCGTACCAGAACGAGTAGCTTG	42	#888888
Frame^{R} 1	AAGGACAGATAACTTTATTTTGCAGTGTAAAGCCTGGGTGCCAGCGGCG	49	#888888
Frame^{R} 2	TAGTTTGTTTTCGCTGAGGCTTGCGCATCGGAGGAAGTCCCT	42	#888888
Frame^{R} 3	CCCTTAGCCGCGTTGGGAAGA	21	#888888
Frame^{R} 4	ATTTTTTCGATACCAGTATAAAGCAAAAGCCACTA	35	#888888
Frame^{R} 5	CTTAATATAAAGTACTAAACGCTAACGAGCACCCT	35	#888888
Frame^{R} 6	TATCAGGGCGATAGCAAGCCGCCCAAAAATAGCAG	35	#888888
Frame^{R} 7	GGAGAATAGCACTTGATGGTGGTTGGCGCTGAGAAAGCGAAAGGATGGC	49	#888888
Frame^{R} 8	ACTGGGGAGATGCATTAATGAATCGG	26	#888888
Frame^{R} 9	ATTTGAATTACAACTTTTTTCATGAACGAGGGTAGCAATATTCGGAACG	49	#888888
Frame^{R} 10	GATGTCAGGAGAACGAGCCACTACGAATACACTATCGTCACCCTCTAAA	49	#888888
Frame^{R} 11	AAAACCCTAAAAAGGAAATAAACAACATGTTAGAA	35	#888888
Frame^{R} 12	ACTTCATAAGAAACGGGAGCAGCGAAAGACAAGGGAGTATAAGAAATTA	49	#888888
Frame^{R} 13	CATCTCGTTTAATTGCGTTCACCGCCTGGCCACAC	35	#888888
Frame^{R} 14	CTTTGAGGACTATCAGAAAATAGTAAAATGTTTGAGA	37	#888888
Frame^{R} 15	ATATAATAAATATTCATTGAATCCTTCCATTGGAACCGATCCGGTGGGT	49	#888888
Frame^{R} 16	AAAGAAGGCACATTCAACGAGGCATAGTAGAAAGATTCATTTAGGAATA	49	#888888
Frame^{R} 17	CGGAGAACGAAAGAACCCCCAAGAGTTACAAAATAACCT	39	#888888
Frame^{R} 18	TAACCGATACGGCTACAGAGG	21	#888888
Frame^{R} 19	GAAATATTATAACGTCTTTCCAGAGCCTAATTTGC	35	#888888
Frame^{R} 20	TCAAACATAAATCAGAGAGATAACACGCCAAGAGAACAGGCCCACGTGG	49	#888888
Frame^{R} 21	TTTAAGGCTTAACTGACCCTTATGAAAACCAATGAGTGAGCTAACTTCC	49	#888888
Frame^{R} 22	CCTTTACAGAGAGAATAAAATGATAGC	27	#888888
Frame^{R} 23	GACAGAGGGTAATTGAGAGAGGCAAAACCAACCCAAATAAGAGTCTGGG	49	#888888

Frame^R 24		CATTCCTGAATCTTACCGATAAGTAATAATCAATC	35	#888888
Frame^R 25		GAAATAAGAGATCATTCTCGAGGTAGTGTTGTTTT	35	#888888
Frame^R 26		CCATATTTAACACCACAA	18	#888888
Frame^R 27		TAACAACAGTAGGGCTTACCGGAAACGTATAAC**/iCy3/**GTG	36	#888888
Frame^R 28		GTACAAAGTCGGGAGAATTAACTGTTATCCCGAACGCGCGGTCAAGGCT	49	#888888
Frame^R 29		AACGCCTGTTTATCAACGACGACAGGACCACACCC	35	#888888
Frame^R 30		GCTTTTAGGCCGCTAATAAACAGGGAAGCGCACGA	35	#888888
Frame^R 31		CGCAAGAAAAAATTTTATAGTTGC	24	#888888
Frame^R 32		ATAAACAATTGAGAATCG	18	#888888
Frame^R 33		TGGGCTAAACAAGTGTTTTTATAATGCGTATCACT	35	#888888
Frame^R 34		CGTTGTTTAGTATCATATGCGTGTATGCGCCAGGGATTTTAGACGCTGC	49	#888888
Frame^R 35		CAGTTTGGGGCAAGAACATTCTAAAATCCAATAAAGGCCGCTTTTGCGG	49	#888888
Frame^R 36		TTAAATTCTGTCCAGACAACGACAAAAGGTAAATATACAAATTCTGCCA	49	#888888
Frame^R 37		GCCAGCACTAGGCTGTCTTAGCGAAACAGCCAACACTCATCTTTGGGCA	49	#888888
Frame^R 38		GAGAGGGAGCTGTAGAAGGGAGGTTAAATCAGCGATTA	38	#888888
Frame^R 39		CTTTCCTCGTTAGGCCACCGAGTAA	25	#888888
Frame^R 40		GCGTAACCGAGCAAGTGTAGCGGTACGGGCACCGA	35	#888888
Frame^R 41		GCGCTAGCCGAAATCTTATAAATCTACGCTGCTCCAAATTTG	42	#888888
Frame^R 42		CCAACGCGCCCAACGTTAATTTCGAACGGTGT	32	#888888
Frame^R 43		GTTTCAGTGAGAATCAGAGCGGGATTGCTTTACCATCAGTACCGCGATA	49	#888888
Frame^R 44		CGCCGGGAAACCTGTCGGTGCCTAAAATAGCAGAC	35	#888888
Frame^R 45		CACGCTCACTGCCCGCTTCACATTACCAGACTGCG	35	#888888
Frame^R 46		GAGCACAGGAACGGTACGCCAGAAGTTTTTCTTCCAGTAAGA	42	#888888
Frame^R 47		AGTCAGGGTGTCCTGAGAGGAGGCCGATTAAGCTA	35	#888888
Frame^R 48		CAGCAGGGCAAGCGACATTATTACAGGTAAGAAAAATCA	39	#888888
Frame^R 49		CATAAAAAAGAAGTTTTG	18	#888888
Frame^R 50		GGCATCATTGAAAGAACTACGTGAGACGAGCTCATAATTACTAGACAAC	49	#888888
Frame^R 51		TATGGAATTACTAATGCAGATACACGTAATGGCGCAGAAGGCGTTTTTC	49	#888888
Frame^R 52		GCACTGAGAGAGTTGCACGAAAATCCTGTAGCTGATTGCCCTTGCCAGT	49	#888888
Frame^R 53		CCAGAGGGGGTAACGAGA	18	#888888
Frame^R 54		TGGATGCTTTAAACAGTAAGACTTGAAAGAGCAAATCAACTCATCCATG	49	#888888
Frame^R 55		GAATCGTCACGCCAAAACATAACCTATACCAAGGGTTGGCCGTAAGCGC	49	#888888
Frame^R 56		CAAATAGCGTCCAATACGACGATACGATTTTTTGGAACCAAGTTTGGCG	49	#888888
Frame^R 57		CCACCAACCTATGTTACGACTTGCACCAATCCCTG	35	#888888
**NBE = Non Blunt End**				
**These staples should create a 4-base loop, thereby preventing stacking**				
NBE10	Ring31′	GCATCAAAACTACAA	15	
NBE9	Ring4′	TGTAGCAATACCGATAGTA	19	
NBE10	Frame^{R}14′	TTGAGGACTATCAGAAAATAGTAAAATGTTTGAGA	35	
NBE9	Frame^{R}18′	TAACCGATACGGCTACAGA	19	
NBE8	Ring7′	ACATTCAACCGATTGAGTCTTAAACAGCTTGTTCC	35	
NBE8	Frame^{R}6′	TATCAGGGCGATAGCAAGCCGCCCAAAAATAGC	33	
NBE7	Ring12′	TGAAAGAAAAAGCGCCAAAGACAAAAG	27	
NBE7	Frame^{R}22′	TTTACAGAGAGAATAAAATGATAGC	25	
NBE6	Ring11′	ACCGAAGCCCTTTTTATAACCT	22	
NBE6	Frame^{R}26′	ATATTTAACACCACAA	16	

NBE5	Ring14'	TCTGTAACTAAATTTATTTTTGCGTAAGTTAGTAATAAC	39
NBE5	Frame^{R}32'	ATAAACAATTGAGAAT	16
NBE4	Ring19'	ATCTCTGGTGCCGGAAACCA	20
NBE4	Frame^{R}42'	AACGCGCCCAACGTTAATTTCGAACGGTGT	30
NBE3	Ring25'	AAGCGCCATTCGCCATTGCTGTTTAGATTTATTGA	35
NBE3	Frame^{R}8'	ACTGGGGAGATGCATTAATGAATC	24
NBE2	Ring28'	AACGAGTCCTGTGTGAAATTGG	22
NBE2	Frame^{R}49'	CATAAAAAAGAAGTTT	16
NBE1	Ring3'	ACAAGTACAAAAGATTACCAATTC	24
NBE1	Frame^{R}53'	AGAGGGGGTAACGAGA	16
**Loops**			
**Staples complementary to the Loops domain**			
Loop1		ATGACCATAAATCAAAAATCAGGTCTTTACCCTGACTATTATAGTCAGAAGCAAAGC	57
Loop2		TTATCCGCTCACAATTCCACACAACATACGAGCCGGAAG	39
Loop3		AAGAGTCTGTCCATCACGCAAATTAACCGTTGTAGCAATACT	42
Loop4		TTGAAATACCGACCGTGTGATAAATAAGGCGTTAAATAAGA	41
Loop5		GAATTGAGTTAAGCCCAATAATAAGAGCAAGAAACAATGAAATAGCAATAGCTATCTTA	59
Loop6		TGCGCCGACAATGACAACAACCATCGCCCACGCA	34
**Ligand Domain = 5 pairs of staples along the Ring/Frame^{R} interface that were modified to contain target-associated sequences (blue) for nucleic acid detection (Fig. 4)**			
Ligand5	Ring4'	ACTCTAAGGGCGGAAGGCTACCTGTAGCAATACCGATAGTA	41
Ligand5	Frame^{R}18'	TAACCGATACGGCTACAGAGGTAGCCTTCCGCCCTT	36
Ligand4	Ring7'	TTGGCATTTGAACACTGCGGCGACATTCAACCGATTGAGTCTTAAACAGCTTGTTCC	57
Ligand4	Frame^{R}6'	TATCAGGGCGATAGCAAGCCGCCCAAAAATAGCAGCCGCAGTGTTCAAAT	50
Ligand3	Ring11'	ACCGAAGCCCTTTTTATAACCTTGTTGGGTCCGTTTGGGTTTGA	44
Ligand3	Frame^{R}26'	CCCAAACGGACCCAACCATATTTAACACCACAA	33
Ligand2	Ring25'	GTGGAGGATGGTCAAGGTGGCAAAGCGCCATTCGCCATTGCTGTTTAGATTTATTGA	57
Ligand2	Frame^{R}8'	ACTGGGGAGATGCATTAATGAATCGGCCACCTTGACCATCC	41
Ligand1	Ring28'	TCTTGTCTCGCCCAGTGTTCCGAACGAGTCCTGTGTGAAATTGG	44
Ligand1	Frame^{R}49'	CATAAAAAAGAAGTTTTGGAACACTGGGCGAGA	33
**Target = Sequences that were detected using OPTIMuS and the Ligand domain.**			
Target5		TAGCCTTCCGCCCTTAGAGT	20
Target4		CCGCAGTGTTCAAATGCCAA	20
Target3		TCAAACCCAAACGGACCCAA	20
Target2		CCACCTTGACCATCCTCCAC	20
Target1		GAACACTGGGCGAGACAAGA	20

Supplementary Note S3: References

1. Ke Y, Bellot G, Voigt NV, Fradkov E, Shih WM. Two design strategies for enhancement of multilayer-DNA-origami folding: underwinding for specific intercalator rescue and staple-break positioning. *Chem Sci* 2011, **3**(8)**:** 2587-2597.

2. Stein IH, Schuller V, Bohm P, Tinnefeld P, Liedl T. Single-Molecule FRET Ruler Based on Rigid DNA Origami Blocks. *Chemphyschem* 2011, **12**(3)**:** 689-695.

3. Woo S, Rothemund PWK. Programmable molecular recognition based on the geometry of DNA nanostructures. *Nature Chemistry* 2011, **3**(8)**:** 620-627.
